# 1104. Characteristics and Seasonality of Children Hospitalized with Parainfluenza Virus Infections (PIV), including PIV-4, New Vaccine Surveillance Network, 2016–2020

**DOI:** 10.1093/ofid/ofad500.077

**Published:** 2023-11-27

**Authors:** Annabelle M de St. Maurice, Yasmeen Z Qwaider, Tess Stopczynski, John V Williams, Marian G Michaels, Leila C Sahni, Julie A Boom, Andrew J Spieker, Eileen J Klein, Janet A Englund, Mary A Staat, Elizabeth P Schlaudecker, Rangaraj Selvarangan, Jennifer E Schuster, Yingtao Zhou, Heidi L Moline, Peter G Szilagyi, Natasha B Halasa, Geoffrey A Weinberg

**Affiliations:** Los Angeles County Department of Public Health, CA; Vanderbilt University Medical Center, Nashville, Tennessee; Vanderbilt University Medical Center, Nashville, Tennessee; University of Pittsburgh, Pittsburgh, Pennsylvania; UPMC Children's Hospital of Pittsburgh, Pittsburgh, Pennsylvania; Baylor College of Medicine and Texas Children’s Hospital, Houston, Texas; Texas Children’s Hospital, Houston, Texas; Vanderbilt University Medical Center, Nashville, Tennessee; University of Washington School of Medicine, Seattle, Washington; Seattle Children’s Hospital, Seattle, Washington; Cincinnati Children’s Hospital Medical Center, Cincinnati, Ohio; Cincinnati Children's Hospital Medical Center, Cincinnati, Ohio; Children’s Mercy Kansas City, Kansas City, Missouri; Children’s Mercy Kansas City, Kansas City, Missouri; National Center for Immunization and Respiratory Diseases U.S. Centers for Disease Control and Prevention, Atlanta, Georgia; Centers for Disease Control and Prevention, Atlanta, Georgia; UCLA School of Medicine, Agoura Hills, California; Vanderbilt University Medical Center, Nashville, Tennessee; University of Rochester School of Medicine & Dentistry, Rochester, NY

## Abstract

**Background:**

Human parainfluenza viruses (PIV) are a leading cause of pediatric acute respiratory infection (ARI), yet limited data exist on PIV-associated hospitalizations, particularly from serotype PIV-4, a serotype reported to cause severe disease. We describe the characteristics and seasonality of PIV-associated ARI hospitalizations, focusing on PIV-4.

**Methods:**

During 12/01/2016-03/31/2020, we enrolled children hospitalized with fever or respiratory symptoms at the 7 children’s hospitals of the CDC’s New Vaccine Surveillance Network. Molecular testing for rhino/enterovirus (RV/EV), respiratory syncytial virus (RSV), influenza virus, PIV (serotypes 1-4), human metapneumovirus, and adenovirus were performed on mid-turbinate nasal or throat specimens collected from enrolled children. Parent interviews and chart abstractions were performed.

**Results:**

A PIV was detected in 1,004 (6%) of 16,282 enrolled children. A PIV and another respiratory virus (most frequently with RV/EV or RSV) were detected in 336 (33%) children; 668 (67%) children tested positive for only 1 PIV (no co-detection of any other virus or co-detected PIV), including PIV-1 (33%), PIV-2 (15%), PIV-3 (40%), and PIV-4 (12%) (Table 1). Among children with only 1 PIV detected, children with PIV-4 infection were older (mean age 3.1 y) and more likely to have an underlying medical condition than children infected with other PIV serotypes. Discharge diagnoses of asthma, otitis media, and pneumonia were most common in children with PIV-4, and croup most common in those with PIV-1 & 2 (Table 2). PIV-1 had biennial fall seasonality; PIV-2 was detected mostly during the fall of 2018; PIV-3 detections peaked annually in the spring; and PIV-4 was detected year-round (Figure). One in 6 children required ICU care.

**Figure:**
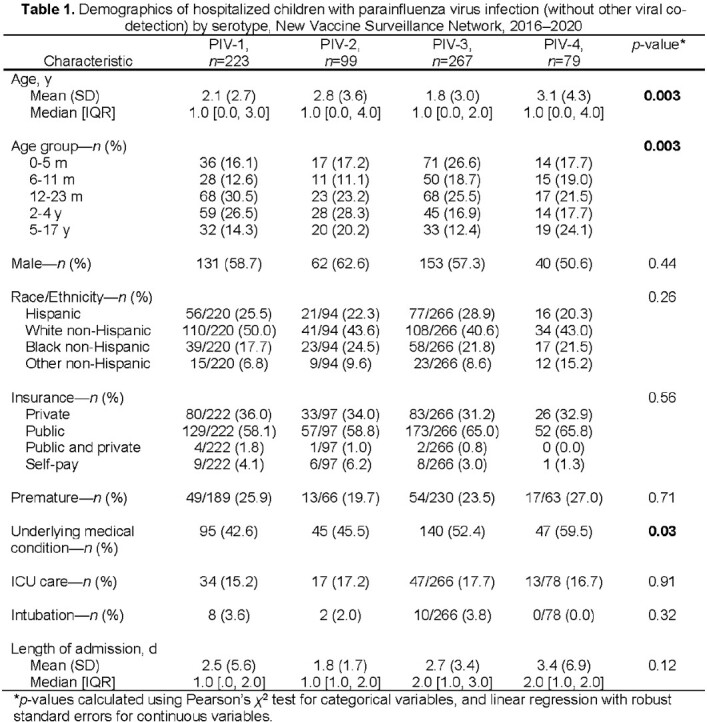

**Figure:**
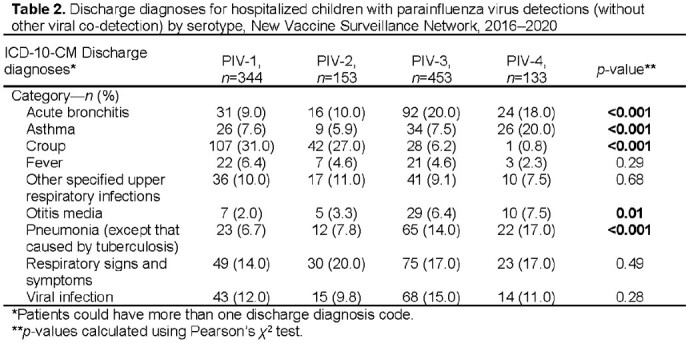

**Figure.**
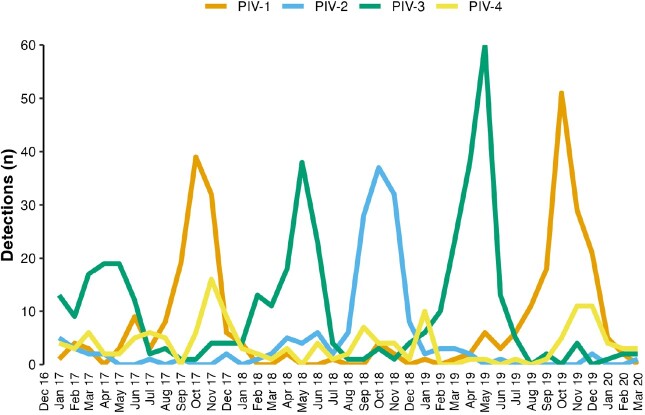
Parainfluenza virus (PIV) detections from hospitalized children by month and PIV serotype, New Vaccine Surveillance Network, 2016–2020

**Conclusion:**

PIV infections were detected in 6% of ARI hospitalizations and occurred throughout the year, with seasonal variation in serotype dominance. Half of children with PIV-related hospitalizations were healthy (without underlying medical conditions). Severe disease (requirement for ICU care) was common in all PIV-associated hospitalizations. Research on PIV vaccines is warranted, with consideration to include PIV-4.

**Disclosures:**

**John V. Williams, MD**, Merck: Grant/Research Support|Quidel: Board Member **Marian G. Michaels, MD, MPH**, Merck: Grant/Research Support|Viracor: Grant/Research Support **Janet A. Englund, MD**, Ark Biopharma: Advisor/Consultant|AstraZeneca: Advisor/Consultant|AstraZeneca: Grant/Research Support|GlaxoSmithKline: Grant/Research Support|Meissa Vaccines: Advisor/Consultant|Merck: Grant/Research Support|Moderna: Advisor/Consultant|Moderna: Grant/Research Support|Pfizer: Advisor/Consultant|Pfizer: Grant/Research Support|Sanofi Pasteur: Advisor/Consultant **Mary A. Staat, MD, MPH**, CDC: Grant/Research Support|Cepheid: Grant/Research Support|Merck: Grant/Research Support|NIH: Grant/Research Support|Pfizer: Grant/Research Support|Up-To-Date: Honoraria **Elizabeth P. Schlaudecker, MD, MPH**, Pfizer: Grant/Research Support|Sanofi Pasteur: Advisor/Consultant **Rangaraj Selvarangan, BVSc, PhD, D(ABMM), FIDSA, FAAM**, Abbott: Honoraria|Altona Diagnostics: Grant/Research Support|Baebies Inc: Advisor/Consultant|BioMerieux: Advisor/Consultant|BioMerieux: Grant/Research Support|Bio-Rad: Grant/Research Support|Cepheid: Grant/Research Support|GSK: Advisor/Consultant|Hologic: Grant/Research Support|Lab Simply: Advisor/Consultant|Luminex: Grant/Research Support **Natasha B. Halasa, MD, MPH**, Merck: Grant/Research Support|Quidell: Grant/Research Support|Quidell: donation of kits|Sanofi: Grant/Research Support|Sanofi: vaccine support **Geoffrey A. Weinberg, MD**, Merck & Co: Honoraria

